# Astrocytes control hippocampal synaptic plasticity through the vesicular-dependent release of D-serine

**DOI:** 10.3389/fncel.2023.1282841

**Published:** 2023-12-07

**Authors:** Daniela Sofia Abreu, Joana I. Gomes, Filipa F. Ribeiro, Maria J. Diógenes, Ana M. Sebastião, Sandra H. Vaz

**Affiliations:** ^1^Faculdade de Medicina, Instituto de Medicina Molecular João Lobo Antunes, Universidade de Lisboa, Lisbon, Portugal; ^2^Faculdade de Medicina, Instituto de Farmacologia e Neurociências, Universidade de Lisboa, Lisbon, Portugal

**Keywords:** astrocyte, gliotransmission, d-serine, synaptic plasticity, tripartite synapse

## Abstract

Astrocytes, the most abundant glial cells in the central nervous system (CNS), sense synaptic activity and respond through the release of gliotransmitters, a process mediated by intracellular Ca^2+^ level changes and SNARE-dependent mechanisms. Ionotropic N-methyl-D-aspartate (NMDA) receptors, which are activated by glutamate along with D-serine or glycine, play a crucial role in learning, memory, and synaptic plasticity. However, the precise impact of astrocyte-released D-serine on neuronal modulation remains insufficiently characterized. To address this, we have used the dominant negative SNARE (dnSNARE) mouse model, which selectively inhibits SNARE-dependent exocytosis from astrocytes. We recorded field excitatory postsynaptic potentials (fEPSPs) in CA3-CA1 synapses within hippocampal slices obtained from dnSNARE mice and wild-type (Wt) littermates. Our results demonstrate that hippocampal θ-burst long-term potentiation (LTP), a critical form of synaptic plasticity, is impaired in hippocampal slices from dnSNARE mice. Notably, this LTP impairment was rescued upon incubation with D-serine. To further investigate the involvement of astrocytes in D-serine-mediated mechanisms of LTP maintenance, we perfused hippocampal slices with L-serine – a substrate used by both neurons and astrocytes for D-serine production. The enhancement in LTP observed in dnSNARE mice was exclusively associated with D-serine presence, with no effects evident in the presence of L-serine. Additionally, both D- and L-serine reduced basal synaptic strength in the hippocampal slices of both Wt and dnSNARE mice. These results provide compelling evidence that distinct processes underlie the modulation of basal synaptic transmission and LTP through D-serine. Our findings underscore the pivotal contribution of astrocytes in D-serine-mediated processes that govern LTP establishment and basal transmission. This study not only provides essential insights into the intricate interplay between neurons and astrocytes but also emphasizes their collective role in shaping hippocampal synaptic function.

## 1 Introduction

Emerging evidence has highlighted the essential role of astrocytes, the most abundant type of glial cell in the brain, as an integral part of tripartite synapses capable of sensing and modifying synaptic activity. Through a repertoire of receptors, ion channels, and transporters expressed on their surfaces, astrocytes sense neighboring neurons’ activity and feedback by releasing gliotransmitters, from which glutamate (Angulo et al., 2004) and ATP (Pascual et al., 2005) have been largely explored. Nonetheless, D-serine has gained significant attention in recent years as a pivotal gliotransmitter (Panatier et al., 2006; Henneberger et al., 2010; Wolosker, 2011; Neame et al., 2019; Koh et al., 2021).

D-serine acts as a co-agonist of N-methyl-D-aspartate (NMDA) receptors (NMDAR), binding specifically to GluN1/GluN2A subunit-containing NMDARs for their complete activation (Mothet et al., 2000; Yang et al., 2003; Shleper et al., 2005; Henneberger et al., 2010; Martineau et al., 2014; Coyle et al., 2020). However, the major site of D-serine synthesis and release remains a subject of ongoing debate. D-serine is synthesized from L-serine by the enzyme serine racemase (SR), which is expressed in both neurons and astrocytes (Williams et al., 2006). Although neurons are traditionally considered the primary producers of D-serine in the brain, astrocytic D-serine appears to be more prevalent than neuronal D-serine in most brain regions (Williams et al., 2006; Martineau et al., 2013, 2014). The “Serine shuttle” concept outlines the key mechanisms governing SR activity and D-serine dynamics in the brain, explaining the conflicting localization patterns of SR and D-serine (Wolosker, 2011). Nonetheless, the hypothesis that D-serine is primarily released by astrocytes still lacks robust evidence. Thus, to investigate the role of vesicular D-serine release by astrocytes in synaptic transmission and plasticity, the transgenic dnSNARE mouse model, with selectively impaired SNARE-dependent gliotransmission, was used. The dnSNARE mice express a dominant-negative form of SNARE proteins in their astrocytes. SNARE proteins are critical for the vesicular release of transmitters. Thus, by interfering with SNARE function in astrocytes, this model allows the assessment of the role of astrocytic gliotransmission (Pascual et al., 2005). The expression of the dominant-negative SNARE proteins can be controlled by adding or removing doxycycline (Dox) from the animals’ drinking water. This provides a high degree of control over when the dominant-negative SNARE proteins are expressed. In addition, astrocytes from dnSNARE mice have been shown to display a 91% reduction in the number of fusion events (Sultan et al., 2015). Thus, the dnSNARE mice is a valuable tool to study astrocytes gliotransmission. Our findings demonstrate that D-serine production and release are necessary when there is higher demand, such as during NMDAR-mediated LTP induction. Indeed, in hippocampal slices from mice with compromised SNARE-dependent gliotransmission, the release of D-serine is compromised, leading to a reduction of LTP, that we were able to rescue upon exposure to D-serine, but not L-serine. Therefore, our study suggests the potential existence of a D-serine shuttle from neurons to astrocytes within the hippocampus. This is corroborated by our electrophysiology data, indicating that D-serine may accumulate in glial vesicles and be released in a SNARE-dependent manner to support the establishment of synaptic plasticity.

## 2 Materials and methods

### 2.1 Animals

**FIGURE 1:**
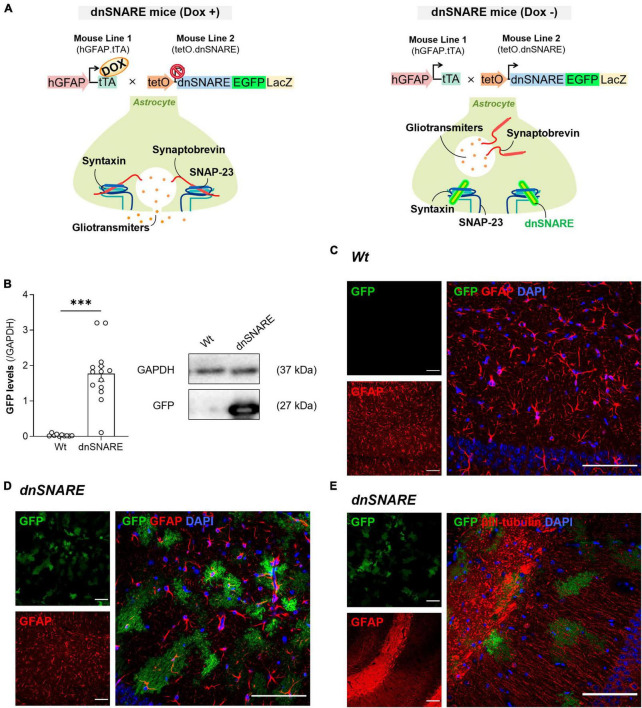
dnSNARE transgene expression is astrocyte-specific. **(A)** The GFAP.tTA mice line carries the human GFAP promoter that drives the expression of tTA, while the tetO.dnSNARE mice line contains a dnSNARE domain, that corresponds to the cytosolic portion of synaptobrevin, along with the EGFP reporter gene, regulated by a tetO operator promotor. Dox administration via drinking water inhibits the transcription of the dnSNARE domain, allowing functional SNARE-dependent release of gliotransmitters. In contrast, in the absence of Dox, the tetO promoter drives the expression of the dnSNARE, resulting in compromised astrocytic vesicular release. **(B)** Western blots of hippocampal slices tissue samples from dnSNARE and Wt mice depict immunoreactive bands for GFP (27 kDa) and GAPDH (loading control; 37 kDa) (Top panel). The histogram shows membrane intensity of the ratio of GFP/GAPDH (*n* = 10–14 animals per group). All values are presented as mean ± S.E.M. from *n* independent observations. Statistical significance was assessed by unpaired *t*-test. Representative confocal images of **(C)** GFP reporter transgene (green) with GFAP (red) and DAPI (blue) in the dorsal CA1 of Wt mice (*n* = 2 mice), of **(D)** GFP reporter transgene (green) with GFAP (red) and DAPI (blue) in the dorsal CA1 of dnSNARE mice (*n* = 2 mice), and **(E)** GFP reporter transgene (green) with βIII-tubulin (red) and DAPI (blue) in the dorsal CA1 of dnSNARE mice (*n* = 2 mice). Scale bars = 100 μm.

All experimental procedures were conducted following the guidelines for the welfare of laboratory animals, according to the guidelines of Directive 2010/63/EU, and the Portuguese Law (DL 113/213). Animals were maintained in controlled temperature (21 ± 1°C) and humidity (55 ± 10%) conditions with a 12:12 h light/dark cycle and access to food and water *ad libitum*. Care was taken to minimize the number of animals sacrificed. For this work, dominant-negative SNARE (dnSNARE) male mice within 8−12 weeks old were used. The generation of dnSNARE mice was performed as previously described by (Pascual et al., 2005). In this strain, the SNARE domain of the synaptobrevin II (dnSNARE) expression is conditionally suppressed in astrocytes, through a tet-Off expression system, allowing the study of SNARE-dependent gliotransmission (Schematic representation on Figure 1A).

#### 2.1.1 Genetic architecture

To generate the dnSNARE mouse model, two different transgenic mouse lines were crossed. The first line comprises of hGFAP.tTA mice, in which the promoter of the astrocyte-specific human glial fibrillary acidic protein (hGFAP) controls the expression of the tetracycline transactivator (tTA). The second line, tetO.dnSNARE, produces reporter genes for enhanced green fluorescent protein (EGFP) and -galactosidase (LacZ), as well as the cytosolic region of the SNARE domain of VAMP2/Synaptobrevin II (amino acids 1−96). Importantly, the tetracycline operator (tetO) regulates the co-expression of these genes in astrocytes.

#### 2.1.2 Conditional control

The administration of doxycycline (Dox) in drinking water (25 L/mL) can turn on or off the expression of dnSNARE, LacZ, and EGFP within astrocytes. Dox attaches to the tTA transgene when it is present, preventing it from binding to the tetO operator. As a result, the dnSNARE, LacZ, and EGFP genes remain suppressed. Therefore, the tTA binds to tetO and initiates the transcription of these genes when Dox is removed from the drinking water. The release of gliotransmitters from astrocytes that are SNARE-dependent is effectively stopped by this system.

#### 2.1.3 Experimental setup

Four to eight weeks before the start of the electrophysiological recordings, Dox was removed from the drinking water of the Wt and dnSNARE mice. It is important to note that to address the impact of Dox removal, Wt (Dox +) and dnSNARE (Dox +) had consistent Dox treatment regimens until electrophysiology recordings day.

### 2.2 *Ex vivo* electrophysiological recordings

For electrophysiological recordings, acute hippocampal slices from both Wt and dnSNARE mice were prepared as previously described (Rei et al., 2020). Mice that were exclusively used *ex vivo* were sacrificed by decapitation after cervical displacement, and the brain was rapidly removed to isolate the hippocampus. The hippocampus was dissected in ice-cold artificial cerebrospinal fluid (aCSF) containing (in mM): 124 NaCl, 3 KCl, 1.2 NaH_2_PO_4_, 25 NaHCO_3_, 2 CaCl_2_, 1 MgSO_4_, and 10 glucose, which was continuously oxygenated with 95% O_2_ and 5% CO_2_. The hippocampal slices were cut perpendicularly to the long axis of the hippocampus (400 μm thick) with a McIlwain tissue chopper. Subsequently, these slices were allowed to recover functionally and energetically for at least 1 h in a resting chamber filled with continuously oxygenated aCSF, at room temperature (RT), before being set up for electrophysiological recordings.

#### 2.2.1 Drug treatment

Hippocampal slices from Wt and dnSNARE mice were allowed to recover for at least 1 h in a resting chamber filled with oxygenated aCSF at RT. The slices were subjected to different conditions: control (no drug incubation), D-serine (10 μM, S4250, Merck, Germany), or L-serine (10 or 50 μM, S4500, Merck, Germany). The compounds were perfused at least 15 min before the start of the recordings.

D-serine, which is a co-agonist of NMDAR (Martineau et al., 2014; Coyle et al., 2020), was previously shown to fully rescue LTP suppression caused by clamping the intra-astrocyte Ca^2+^ levels, when used at a concentration of 10 μM (Henneberger et al., 2010). Additionally, to explore the potential role of astrocytes in D-serine release using L-serine as a substrate, L-serine was applied in the perfusion bath at the same concentration as D-serine (10 μM) and at a known concentration (50 μM) that rescues LTP in animals conditionally lacking Pgdh in astrocytes (Le Douce et al., 2020).

#### 2.2.2 Extracellular recordings of field excitatory postsynaptic potentials (fEPSPs)

Following the slice recovery time, hippocampal slices were transferred to a recording chamber for submerged slices (1 mL capacity plus 5 mL dead volume) and were constantly superfused at a flow rate of 3 mL/min with aCSF kept at 32°C and gassed with 95% O_2_ and 5% CO_2_. fEPSPs were recorded extracellularly by placing a microelectrode (4−8 MΩ resistance), filled with aCSF solution, in the *stratum radiatum* of the CA1 area. Stimulation was delivered through a bipolar concentric wire electrode fabricated from platinum/iridium wire (25 μm diameter,<800 kΩ impedance) positioned in the *Schaffer collaterals*. An extracellular recording electrode was placed among the apical dendrites of the CA1 pyramidal cells, and stimulating electrodes were positioned in the *Schaffer collaterals* to stimulate two separate input pathways. fEPSPs were evoked by the stimulation of the two different pathways of the *Schaffer collateral* fibers every 10 s (rectangular pulses of 0.1 ms duration). Averages of eight consecutive responses were continuously acquired using an Axoclamp-2B amplifier (Axon Instruments, Foster City, CA, USA), digitized with the WinLTP program (Anderson and Collingridge, 2001), and quantified as the slope of the initial phase of the averaged fEPSPs. The stimulus intensity was adjusted at the beginning of the experiment to obtain a fEPSP slope close to 0.5 mV/ms, to evoke fEPSPs of amplitude about 50% of maximal amplitude with minimal contamination by a population spike. fEPSPs were recorded under basal stimulation conditions (standard stimulus intensity and frequency) and the stability of fEPSP slope values was monitored for more than 20 min before starting the LTP protocol. Two or three slices per animal were tested on each experimental day (under different experimental conditions). dnSNARE electrophysiological characterization: (1) LTP: Wt *n* = 5 vs. dnSNARE *n* = 5; (2) PPF: Wt *n* = 4 vs. dnSNARE *n* = 4; (3) I/O curve: Wt *n* = 4 vs. dnSNARE *n* = 4. Effect of D-serine 10 μM: (1) LTP: Wt *n* = 5 vs. Wt + D-serine 10 μM *n* = 9; dnSNARE *n* = 5 vs. dnSNARE + D-serine 10 μM *n* = 7; (2) PPF: Wt *n* = 4 vs. Wt + D-serine 10 μM *n* = 8; dnSNARE *n* = 4 vs. dnSNARE + D-serine 10 μM *n* = 9; (3) I/O curve: Wt *n* = 4 vs. Wt + D-serine 10 μM *n* = 7; dnSNARE *n* = 4 vs. dnSNARE + D-serine 10 μM *n* = 7. Effect of L-serine 10 and 50 μM: (1) LTP:Wt *n* = 6 vs. Wt + L-serine 10 μM *n* = 8 vs. Wt + L-serine 50 μM *n* = 7; dnSNARE *n* = 6 vs. dnSNARE + L-serine 10 μM *n* = 8 vs. dnSNARE + L-serine 50 μM *n* = 7; (2) PPF: Wt *n* = 4 vs. Wt + L-serine 10 μM *n* = 7 vs. Wt + L-serine 50 μM *n* = 7; dnSNARE *n* = 6 vs. dnSNARE + L-serine 10 μM *n* = 6 vs. dnSNARE + L-serine 50 μM *n* = 6; I/O curve: Wt *n* = 6 vs. Wt + L-serine 10 μM *n* = 6 vs. Wt + L-serine 50 μM *n* = 4; dnSNARE *n* = 6 vs. dnSNARE + L-serine 10 μM *n* = 6 vs. dnSNARE + L-serine 50 μM *n* = 4. The traces shown from the representative experiments are composed of stimulus artifact, presynaptic fiber volley, and fEPSP.

#### 2.2.3 Paired-pulse facilitation (PPF) recordings

Paired-pulse facilitation (PPF) is a type of short-term synaptic plasticity that depends on the transmitter release probability during a second stimulus. It results from residual Ca^2+^ near active zones or a lasting effect of Ca^2+^ on a Ca^2+^ sensor (Chen et al., 2004). Thus, when two stimuli are delivered within a short interval, the response invoked by the second stimulus can be either enhanced or depressed relative to the response invoked by the first stimulus (Zucker and Regehr, 2002). We measured PPF as the ratio of the slopes of two consecutive fEPSPs (fEPSP1/fEPSP0) elicited with a 50 ms interstimulus interval, with each pair delivered every 15 s. We averaged the results of six paired-pulse responses to calculate the PPF ratio for each slice.

#### 2.2.4 Input/Output (I/O) curves

After establishing a stable baseline with standard stimulation, we decreased the stimulus to 60 μA, below the threshold for evoking an fEPSP, and then increased it in 20 μA steps (from 60 to 320 μA). For each stimulation intensity, data from two consecutive averaged fEPSPs was collected. The Input/Output (I/O) curves were plotted as the fEPSP slope against the stimulus intensity, which provides a measure of basal synaptic transmission (Diógenes et al., 2011). The top parameter, representing the maximum slope values, was determined by extrapolation upon non-linear fitting of the I/O curve.

#### 2.2.5 Long-term Potentiation (LTP) induction

Long-term potentiation (LTP) was induced after maintaining a stable recording of the fEPSP slope for at least 30 min. We used a θ-burst stimulation LTP-inducing protocol, involving 1 stimulus with 4 bursts separated by 200 ms, with each burst consisting of 4 pulses at 100 Hz. θ-burst stimulation is chosen for its physiological relevance to learning and memory in the hippocampus (Albensi et al., 2007). The stimulus intensity remained constant during data acquisition. LTP magnitude was quantified as the percentage change in the average fEPSP slope measured 50−60 min after LTP induction, compared to the average slope measured 15 min before LTP induction.

#### 2.2.6 Post-tetanic potentiation (PTP) recordings

Post-tetanic potentiation (PTP), is an enhancement of transmitter release on a minute time scale due to residual Ca^2+^ in the neuronal terminal during high-frequency firing periods (Zucker and Regehr, 2002; Korogod et al., 2007). We quantified PTP by averaging the fEPSP slope obtained in the first 4 min after LTP induction (Habets and Borst, 2007).

### 2.3 Western blot

Western blot was used to assess the levels of EGFP (dnSNARE transgene) (Sardinha et al., 2017). After the electrophysiological recordings, tissue homogenates were prepared from the remaining hippocampal slices (Wt: *n* = 10, dnSNARE: *n* = 14). Samples were homogenized by sonication, in Radio Immuno Precipitation Assay lysis buffer [1% Non-idet^®^ P40 Substitute (NP40), 50 mM Tris-HCl (pH 7.5), 150 mM NaCl, 5 mM ethylenediamine tetra-acetic acid, 0.1% sodium dodecyl sulfate (SDS) and 1% Triton X-100] containing one protease inhibitor cocktail tablet (Roche, Germany) for each 10 mL. Lysates were centrifuged (13000 g, 10 min) and the supernatant was collected. The supernatant protein concentration was determined using a commercially available Bradford Assay kit (Bio-Rad Laboratories, CA, USA). Equal quantities (50 μg) of the prepared protein samples were loaded and separated using 10% SDS-polyacrylamide gel electrophoresis and transferred to a polyvinylidene fluoride membrane (GE Healthcare, Buckinghamshire, UK). NZYColour Protein Marker II (NZYTech, Lisbon, Portugal) was used as a protein molecular weight marker. Protein transfer efficacy was confirmed by Ponceau S staining. Membranes were blocked in 3% bovine serum albumin in Tris-buffered saline with-Tween (20 mM Tris base, 137 mM NaCl and 0.1% Tween-20) (TBST-T) for 1 h. Membranes were incubated overnight at 4°C, with anti-GFP rabbit polyclonal, Alexa Fluor 488 (1:1000, #A-21311 Invitrogene, USA), and anti-glyceraldehyde 3-phosphate dehydrogenase (GAPDH) mouse monoclonal (1:5000, AM4300, Life Technologies, USA). Goat anti-mouse IgG-horseradish peroxidase-conjugated (1:10 000, 1706516, Bio-Rad, USA) secondary antibody was incubated for 1 h at RT. After washing, proteins were revealed with ClarityTM Western ECL Substrate (Bio-Rad, USA), using a ChemiDocTM XRS + imaging system with Image LabTM software (Bio-Rad, USA). Band quantification was performed using the Image Lab software (Bio-Rad, USA), and all samples were normalized to the loading control GAPDH). After using anti-GFP, the membranes were subjected to the stripping protocol to reprobe with a anti-GAPDH antibody. The membrane, with the protein side facing up, was incubated in the stripping solution. This incubation was carried out at RT with gentle agitation and lasted for 10−20 min. Post-incubation in the stripping solution (25 mM glycine-HCl and 1% SDS, with pH 2), the membranes were washed three times with TBS-T for 5 min.

The representative image from Figure 1B is a small excerpt from Supplementary Figure 1. The images were not manipulated or altered in any way that could misrepresent the experimental findings.

### 2.4 Immunofluorescence assay

To visualize the expression of the dnSNARE transgene reporter (EGFP) and its co-expression with astrocytic and neuronal markers, immunohistochemistry was performed on brain slices from Wt and dnSNARE mice. Mice were deeply anesthetized with 100 μL of pentobarbital, by intraperitoneal injection, and readily perfused transcardially with cold aCSF solution. After perfusion, mice were decapitated, brains were removed, and post-fixed by immersion in 4% PFA overnight at 4°C. After a quick wash in PBS, the brains were immersed in a 15% sucrose solution at 4°C, and once in the bottom of the tube, they were changed to a 30% sucrose solution. Coronal brain sections (40 μm thick) were obtained using a vibratome (Leica, Germany), and stored at 4°C in PBS with 0.02% sodium azide until used. The antibodies staining protocol started with three washes with PBS, for 10 min each, followed by permeabilization for 30 min (RT) with 5% Triton X-100 in PBS. The slices were then rinsed in PBS and incubated with 10% fetal bovine serum in PBS blocking solution (to reduce non-specific binding) for 30 min at RT. After blocking, slices were incubated with the following primary antibodies, diluted in the blocking solution, at 4°C overnight: anti-GFAP rabbit polyclonal (1:750, Sigma-Aldrich, Germany) and anti-βIII tubulin rabbit monoclonal (1:500, Sigma-Aldrich, Germany). Next, brain slices were rinsed three times in PBS and then incubated with the respective species-specific secondary antibodies: Alexa Fluor^®^ 568 donkey anti-rabbit and Alexa Fluor^®^ 647 donkey anti-rabbit (1:500, Invitrogen, USA) in PBS, for 2 h, in the dark at RT. After rinsing the brain slices with PBS, the nucleic acids were stained with DAPI (4’,6’-diamidino-2-phenylindole, 1:1000, Invitrogen, USA) for 7 min in the dark. After washing with PBS (3 × 10 min), slices were mounted on microscope slides (SuperfrostTM Plus, ThermoFisher Scientific, USA) with Mowiol (non-absorbing compound without autofluorescence and light scattering), and glass coverslips on top, and allowed to dry for at least 24 h. Images of the CA3-CA1 hippocampal regions were captured using a Zeiss LSM 880 with an Airyscan (Carl Zeiss, Germany) confocal point-scanning microscope, with 20 x and 40 x objectives, and analyzed using FIJI open-source software. Each set of images, of the same condition, was composed of 3-4 slices from two animals, separated from each other by 240 μm.

## 3 Results

### 3.1 The transgene expression in the dnSNARE mouse is astrocyte-specific

Despite being a widely used model, the effectiveness of the dnSNARE mouse model as a good model to study SNARE-dependent vesicular release of astrocytic gliotransmitters was questioned (Fujita et al., 2014). Although efforts have been made to produce evidence to support this model (Pascual et al., 2005; Sultan et al., 2015; Sardinha et al., 2017; Lalo et al., 2021), we performed complementary experiments to revalidate the dnSNARE mouse model, and reaffirm the specificity of astrocytic “SNARE-positive cells.” Thus, highlight astrocytes contribution to synapse function through SNARE-dependent release of gliotransmitters.

As previously reported, dnSNARE mice harbor the Lac-Z, EGFP, and dnSNARE genes in their astrocytes, and upon removal of Doxycycline (Dox) from their drinking water, they initiate the expression of the dnSNARE domain (Figure 1A). Since dnSNARE protein levels are difficult to detect in brain samples, as the available antibodies detect both exogenous and endogenous forms of synaptobrevin II, dnSNARE transgene protein levels were indirectly screened by EGFP quantification. This approach, as established in the work of (Sardinha et al., 2017), is based on the direct correlation between EGFP levels and dnSNARE transgene expression. This investigation aimed to underscore the astrocytic specificity of the dnSNARE protein expression. The results indicate that the transgene is specific for dnSNARE mice (Wt: 0.033 ± 0.010, dnSNARE: 1.77 ± 0.212, unpaired Student’s *t*-test: *t* = 6.889, ***p ≤ 0.001, Wt *n* = 10, dnSNARE *n* = 14, Figure 1B, Supplementary Figure 1), supporting the genotyping data. Moreover, it was also observed that GFP has a variable expression across dnSNARE mice.

Furthermore, to confirm the astrocyte-specificity of transgene expression, we conducted immunofluorescence staining on brain slices containing the hippocampus from Wt and dnSNARE mice. Taking advantage of the innate EGFP fluorescence, antibodies to label astrocytic or neuronal markers were used to appraise co-localization. Immunohistochemical analysis confirmed that EGFP co-localized with GFAP-positive astrocytes within the dorsal CA1 area of dnSNARE mice (*n* = 2 mice), whereas its presence was notably absent in the same region of the hippocampus in Wt mice (*n* = 2 mice) (Figure 1C). Moreover, to further validate astrocyte specificity and to eliminate the possibility of neuronal expression of the dnSNARE transgene, immunostaining with βIII-tubulin, which stains neurites, was performed. The staining failed to show an overlap between EGFP and βIII-tubulin in the CA1 area of dnSNARE mice (*n* = 2 mice) (Figure 1D). Therefore, combined with previous reports (Pascual et al., 2005; Halassa et al., 2009; Sardinha et al., 2017), these findings provide robust support for astrocyte-specific dnSNARE expression and exclude the potential neuronal expression of dnSNARE transgene.

### 3.2 Compromised gliotransmission impairs LTP

**FIGURE 2:**
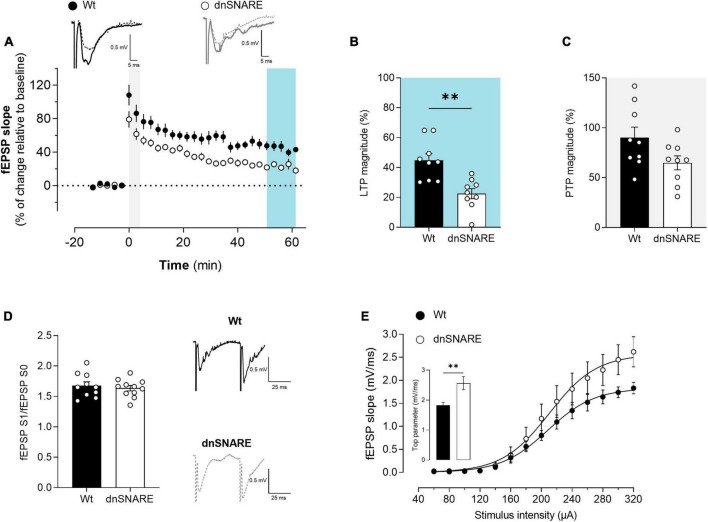
Blocking astrocytic gliotransmission decreases LTP. **(A)** Time course of changes in fEPSP slope after θ-burst stimulation in hippocampal slices from Wt (*n* = 5) and dnSNARE mice (*n* = 5). Representative traces of fEPSPs before (dashed line) and after (bold line) stimulation are displayed above. Scale bar: 5 ms (horizontal), 0.5 mV (vertical). The blue highlighted column represents LTP (B), while the gray highlighted column corresponds to PTP (C). **(B)** Comparison of the magnitude of LTP induced by the θ-burst stimulation. **(C)** Comparison of PTP magnitude. **(D)** PPF ratio obtained from Wt (*n* = 4) and dnSNARE mice (*n* = 4). On the left are illustrated representative tracings of PPF obtained from slices from Wt (bold line) and dnSNARE mice (dashed line). Scale bar: 25 ms (horizontal), 0.5 mV (vertical). **(E)** I/O curves showing how fEPSP slope changes with different stimulation intensities (60–320 μA) in hippocampal slices from Wt (*n* = 4) and dnSNARE mice (*n* = 4). All values are presented as mean ± S.E.M. of *n* independent experiments. Statistical significance was assessed by unpaired *t*-test.

To study the role of SNARE-dependent gliotransmission in hippocampal synaptic plasticity, LTP at CA3-CA1 synapses was elicited by delivering a θ-burst stimulation paradigm to hippocampal slices from Wt and dnSNARE mice. θ-burst stimulation of hippocampal slices from Wt and dnSNARE mice led to an initial enhancement of the fEPSP slope followed by a decrease and stabilization period. At the end of the recording interval (50−60 min after θ-burst), the fEPSP slope values remained higher than before θ-burst stimulation (Figure 2A). LTP magnitude is significantly smaller in dnSNARE mice in comparison to Wt mice (Wt: 47.4 ± 7.65, dnSNARE: 23.1 ± 6.05, unpaired Student’s *t*-test: *t* = 2.48, *p = 0.038, Wt: *n* = 5, dnSNARE: *n* = 5, Figure 2B). Besides, the dnSNARE expression was found to not alter PTP (Wt: 107.8 ± 13.3, dnSNARE: 69.0 ± 10.9, unpaired Student’s *t*-test: *t* = 2.25, *p* = 0.055, Wt: *n* = 5, dnSNARE: *n* = 5, Figure 2C). Overall, these results are indicative of astrocytic regulation, through SNARE-dependent release of gliotransmitters, of the strength of long-term forms of synaptic plasticity, as previously reported (Pascual et al., 2005).

### 3.3 Presynaptic function is not modulated by gliotransmission

To test for putative presynaptic modifications in the hippocampus of dnSNARE mice, the short-term form of plasticity PPF was assessed by eliciting two consecutive fEPSPs (fEPSP1/fEPSP0) with a 50 ms interstimulus interval, each pair being delivered once every 15 s. When comparing the ratio between the second and first fEPSP slope values (fEPSP1/fEPSP0), no differences were detected in hippocampal slices from Wt and dnSNARE mice (Wt: 1.71 ± 0.09, dnSNARE: 1.58 ± 0.07, unpaired Student’s *t*-test: *t* = 1.096, *p* = 0.315, Wt: *n* = 4, dnSNARE: *n* = 4, Figure 2D). These results demonstrate that blocking of gliotransmission does not interfere with presynaptic mechanisms associated with the release of neurotransmitters to the synapse.

### 3.4 Basal synaptic transmission is enhanced in animals with impaired gliotransmission

To investigate the role of astrocytic signaling in the modulation of basal synaptic transmission, I/O curves were recorded from acute hippocampal slices prepared from Wt and dnSNARE mice, as described in the methods. Differences in the synaptic response to different stimulations were observed between Wt and dnSNARE mice tested (Figure 2E). Animals with impaired gliotransmission display a higher top parameter, which is the maximal value obtained by extrapolation upon non-linear fitting of the fEPSP slope against stimulus intensity (Wt: 1.68 ± 0.06, dnSNARE: 2.68 ± 0.26, unpaired Student’s *t*-test: *t* = 3.76, ** *p* = 0.0095, Wt: *n* = 4, dnSNARE: *n* = 4, Figure 2E). These results suggest that the blockade of vesicular gliotransmitter release in astrocytes increases the synaptic responses to strong stimulation, leading to an enhancement of basal synaptic transmission, as previously reported (Pascual et al., 2005).

To ensure that the observed effects were indeed induced by the removal of Dox, that induce SNARE transgene expression, we conducted fEPSP recordings on hippocampal slices of dnSNARE mice that had Dox in their drinking water until recordings’ day [here labeled as “dnSNARE (Dox +)”]. It is important to note that all Wt animals included in the experiments were subject to the same Dox treatment regimen, with Dox provided in their drinking water [Wt (Dox +)]. This control experiment allowed us to compare the impact of Dox removal specifically in the context of driving the dnSNARE mice outcomes.

No differences in the synaptic response to different stimulations were observed between Wt (Dox +) and dnSNARE (Dox +) mice tested [Wt (Dox +): 2.28 ± 0.167, dnSNARE (Dox +): 2.26 ± 0.122, unpaired Student’s *t*-test: *t* = 0.131, *p* = 0.900, Wt (Dox +): *n* = 4, dnSNARE (Dox +): *n* = 4, Supplementary Figures 2A, D]. Additionally, θ -burst stimulation of hippocampal slices from Wt and dnSNARE mice led to an initial enhancement of the fEPSP slope followed by a decrease and stabilization period. At 50−60 min after θ-burst, the fEPSP slope values remained higher than before θ-burst stimulation (Supplementary Figure 2A). LTP magnitude did not differ from dnSNARE mice and Wt mice [Wt (Dox +): 44.7 ± 11.1, dnSNARE (Dox +): 44.1 ± 6.97, unpaired Student’s *t*-test: *t* = 0.047, *p* = 0.963, Wt (Dox +): *n* = 5, dnSNARE (Dox +): *n* = 5, Supplementary Figure 2B]. PTP also remained unaltered in the presence of Dox [Wt (Dox +): 79.8 ± 5.83, dnSNARE (Dox +): 68.8 ± 6.76, unpaired Student’s *t*-test: *t* = 1.239, *p* = 0.251, Wt (Dox +): *n* = 5, dnSNARE (Dox +): *n* = 5, Supplementary Figure 2C]. Moreover, These results indicate that dnSNARE (Dox +) mice do not exhibit significant differences from Wt (Dox +) mice. Therefore, these results strongly confirmed that the observed effects of dnSNARE animals are primarily attributed to the absence of Dox that leads to dnSNARE transgene expression that will prevent the SNARE dependent gliotransmitters release.

### 3.5 D-serine rescues LTP impairments in animals with impaired gliotransmission

D-serine, synthesized from L-serine, is a well-established NMDAR co-agonist that lacks robust evidence of its primary source (Coyle et al., 2020). Thus, we next investigated the role of astrocytic D-serine in basal synaptic transmission and plasticity on the transgenic dnSNARE mice and their respective controls. Accordingly, fEPSPs were recorded, θ-burst stimulation was used to induce LTP and evaluate synaptic plasticity, I/O curve protocol was performed to assess basal synaptic transmission and PPF protocol was induced to analyze pre-synaptic activity in the presence of D-serine (10 μM) and L-serine (10 and 50 μM).

**FIGURE 3:**
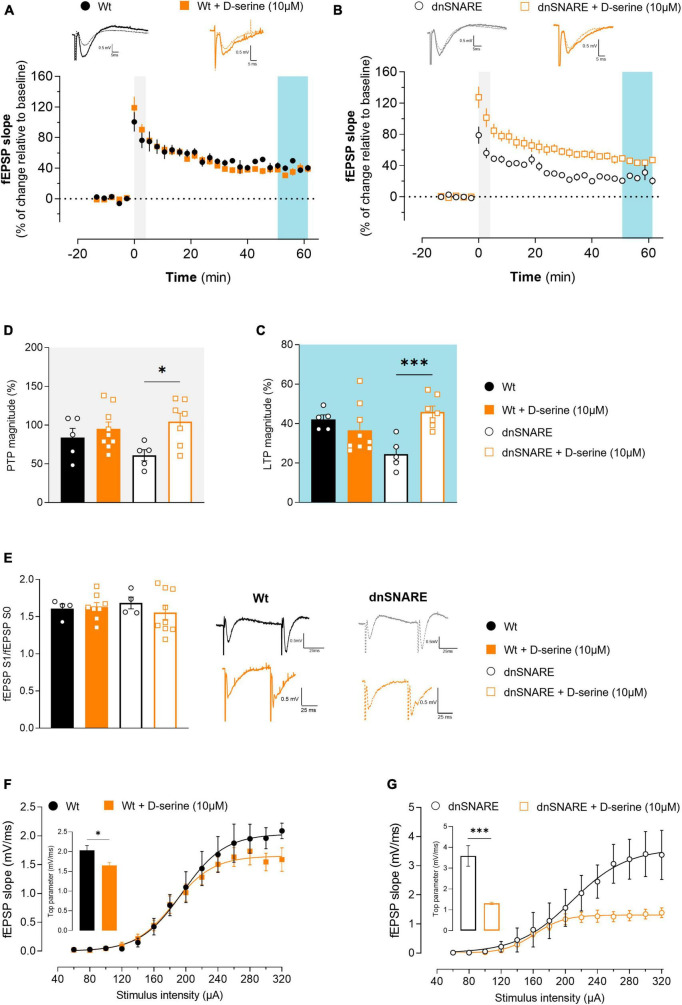
D-serine decreases LTP in Wt animals while increasing PTP and decreasing basal synaptic transmission in animals with compromised gliotransmission. **(A)** Time course of changes in fEPSP slope after θ-burst stimulation in hippocampal slices from Wt mice perfused with D-serine (*n* = 9) with respective controls (*n* = 5). The blue highlighted column represents LTP (C), while the gray highlighted column corresponds to PTP (D). Representative traces of fEPSPs before (dashed line) and after (bold line) stimulation are displayed above. Scale bar: 5 ms (horizontal), 0.5 mV (vertical). **(B)** Time course of changes in fEPSP slope after θ-burst stimulation in hippocampal slices from dnSNARE mice perfused with D-serine (*n* = 7) with respective controls (*n* = 5). Representative traces of fEPSPs before (dashed line) and after (bold line) stimulation are displayed above. **(C)** Comparison of the magnitude of LTP induced by the θ-burst stimulation in the presence and absence of D-serine for Wt and dnSNARE perfused hippocampal slices. **(D)** Comparison of PTP magnitude induced by the θ-burst stimulation in the presence and absence of D-serine for Wt and dnSNARE perfused hippocampal slices. **(E)** PPF ratio obtained from Wt (Wt controls *n* = 4, Wt + D-serine 10 μM *n* = 8) and dnSNARE mice (dnSNARE controls *n* = 4, dnSNARE + D-serine 10 μM *n* = 9). On the left are illustrated representative tracings of PPF obtained from slices from Wt (bold line) and dnSNARE mice (dashed line) in the presence and absence of D-serine. Scale bar: 25 ms (horizontal), 0.5 mV (vertical). **(F)** I/O curves derived from Wt mice hippocampal slices perfused with D-serine (*n* = 7) or without (*n* = 4) showing how fEPSP slope changes with different stimulation intensities (60–320 μA). **(G)** I/O curves derived from dnSNARE mice hippocampal slices perfused with D-serine (*n* = 7) or without (*n* = 4). All values are presented as mean ± S.E.M. of *n* independent experiments. Statistical significance was assessed by unpaired *t*-test.

To unveil the effects of D-serine in synaptic plasticity, LTP at CA3-CA1 synapses was elicited by delivering a θ-burst stimulation paradigm to hippocampal slices from Wt (Figure 3A) and dnSNARE (Figure 3B) mice after D-serine (10 μM) perfusion, at least 15 min before LTP induction. D-serine did not affect LTP magnitude in hippocampal slices from Wt mice (Wt: 42.1 ± 2.28, Wt + D-serine 10 μM: 36.6 ± 4.07, unpaired Student’s *t*-test: *t* = 0.945, *p* = 0.363, Wt: *n* = 5, Wt + D-serine 10 μM: *n* = 9, Figure 3C). When the same paradigm of θ-burst stimulation protocol was applied after perfusion of D-serine in slices from dnSNARE mice, the LTP magnitude significantly increased when compared to dnSNARE slices not treated with D-serine (dnSNARE: 24.6 ± 3.54, dnSNARE + D-serine 10 μM: 45.9 ± 2.98, unpaired Student’s *t*-test: *t* = 4.620, ***p = 0.001, dnSNARE: *n* = 5, dnSNARE + D-serine: 10 μM *n* = 7, Figure 3C), and reached values similar to the LTP magnitude of Wt mice.

Furthermore, the presence of D-serine did not alter PTP magnitude for Wt (Wt: 83.9 ± 11.9, Wt + D-serine 10 μM: 95.2 ± 8.91, unpaired Student’s *t*-test: *t* = 0.756, *p* = 0.464, Wt *n* = 5, Wt + D-serine 10 μM *n* = 9, Figure 3D), but noticeably significantly increase it for dnSNARE mice (dnSNARE: 61.243 ± 7.13, dnSNARE + D-serine 10 μM: 104.6 ± 10.9, unpaired Student’s *t*-test: *t* = 3.014, *p = 0.013, dnSNARE *n* = 5, dnSNARE + D-serine 10 μM *n* = 7, Figure 3D). In summary, in mice with compromised gliotransmission, D-serine significantly affects short-term forms of synaptic plasticity while recovering LTP induction to control levels. Thus, these findings suggest a critical role of astrocytes in the tight regulation of D-serine molecule release, with impact on LTP.

### 3.6 Presynaptic function is not modulated by D-serine

To detect presynaptic modifications caused by exogenous D-serine, the short-term form of plasticity PPF was assessed by eliciting two consecutive fEPSPs (fEPSP1/fEPSP0). When comparing the ratio between the second and first fEPSP slope values (fEPSP1/fEPSP0) with and without D-serine, no differences were detected in hippocampal slices from Wt (Wt: 1.61 ± 0.062, Wt + D-serine 10 μM: 1.63 ± 0.06, unpaired Student’s *t*-test: *t* = 0.202, *p* = 0.844, Wt: *n* = 4, Wt + D-serine 10 μM: *n* = 8, Figure 3E) nor dnSNARE mice (dnSNARE: 1.68 ± 0.08, dnSNARE + D-serine 10 μM: 1.56 ± 0.10, unpaired Student’s *t*-test: *t* = 0.830, *p* = 0.424, dnSNARE: *n* = 4, dnSNARE + D-serine 10 μM: *n* = 9, Figure 3E). Thus, these results demonstrate that D-serine does not interfere with presynaptic mechanisms associated with the release of neurotransmitters to the synapse.

### 3.7 D-serine decreases basal synaptic transmission

To analyze the modulatory role of D-serine on basal synaptic transmission, I/O curves were recorded from acute hippocampal slices perfused with or without D-serine. In hippocampal slices of Wt mice, D-serine decreases the top parameter (Wt: 2.04 ± 0.12, Wt + D-serine 10 μM: 1.65 ± 0.07, unpaired Student’s *t*-test: *t* = 2.875, *p = 0.018, Wt *n* = 4, Wt + D-serine 10 μM *n* = 7, Figure 3F). Moreover, the same was observed in hippocampal slices of dnSNARE mice (dnSNARE: 3.59 ± 0.45, dnSNARE + D-serine 10 μM: 1.31 ± 0.06, unpaired Student’s *t*-test: *t* = 6.194, ***p = 9.0002, dnSNARE *n* = 4, dnSNARE + D-serine 10 μM *n* = 7, Figure 3G). These results suggest that D-serine modulates basal synaptic transmission through a distinct process from LTP.

### 3.8 L-serine does not alter LTP in animals with compromised gliotransmission

Considering that D-serine is biosynthesized from L-serine, hippocampal slices from Wt and dnSNARE mice were perfused with 10 or 50 μM of L-serine to assess if, in the absence of gliotransmission, L-serine produces the same effects as D-serine.

**FIGURE 4:**
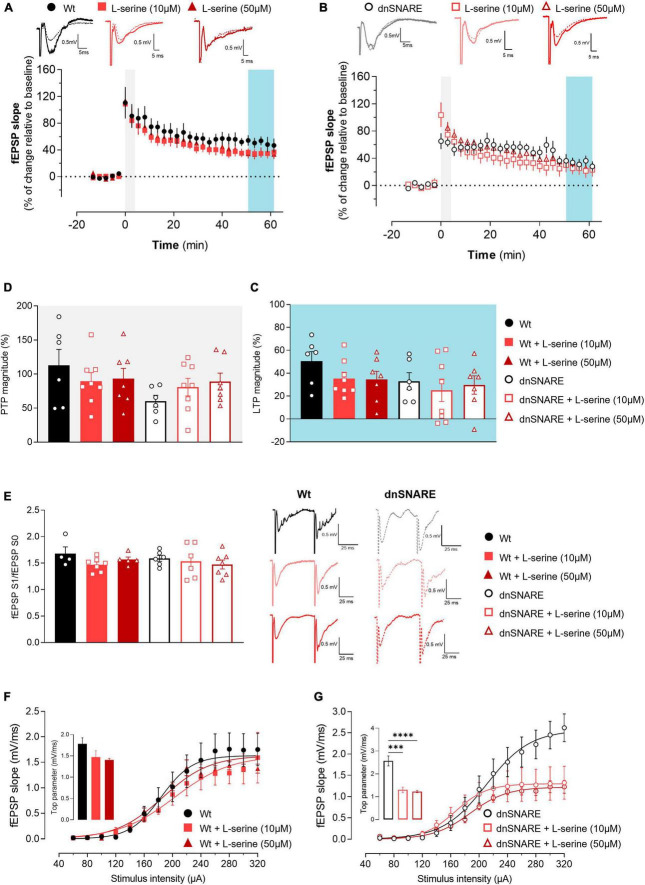
L-serine does not affect LTP nor short forms of synaptic plasticity but increases basal synaptic transmission in animals with compromised gliotransmission. **(A)** Time course of changes in fEPSP slope after θ-burst stimulation in hippocampal slices from Wt mice perfused with 10 μM (*n* = 8) and 50 μM of L-serine (*n* = 7) with respective controls (*n* = 6). The blue highlighted column represents LTP (C), while the gray highlighted column corresponds to PTP (D). Representative traces of fEPSPs before (dashed line) and after (bold line) stimulation are displayed above. Scale bar: 5 ms (horizontal), 0.5 mV (vertical). **(B)** Time course of changes in fEPSP slope after θ-burst stimulation in hippocampal slices from dnSNARE mice perfused with 10 μM (*n* = 8) and 50 μM of L-serine (*n* = 7), and the respective controls (*n* = 6). Representative traces of fEPSPs before (dashed line) and after (bold line) stimulation are displayed above. **(C)** Comparison of the magnitude of LTP induced by the θ-burst stimulation in the presence and absence of L-serine for Wt and dnSNARE perfused hippocampal slices. **(D)** Comparison of PTP magnitude induced by the θ-burst stimulation in the presence and absence of L-serine for Wt and dnSNARE perfused hippocampal slices. **(E)** PPF ratio obtained without (Wt, *n* = 4; dnSNARE, *n* = 6) and after perfusion with 10 μM (Wt, *n* = 7; dnSNARE, *n* = 6) and 50 μM (Wt, *n* = 5; dnSNARE, *n* = 7) of L-serine. On the left are illustrated representative tracings of PPF obtained from slices from Wt (bold line) and dnSNARE mice (dashed line) in the presence and absence of the different concentrations of L-serine. Scale bar: 25 ms (horizontal), 0.5 mV (vertical). **(F)** I/O curves derived from Wt mice hippocampal slices perfused with 10 μM of L-serine (*n* = 6), 50 μM of L-serine (*n* = 4), and the respective controls (*n* = 6) showing how fEPSP slope changes with different stimulation intensities (60–320 μA). **(G)** I/O curves derived from dnSNARE mice hippocampal slices perfused with 10 μM of L-serine (*n* = 6), 50 μM of L-serine (*n* = 4), and the respective controls (*n* = 6) All values are presented as mean ± S.E.M. of *n* independent experiments. Statistical significance was assessed by One-way ANOVA followed by Holm-Sidak’s *post-hoc* test for multiple comparisons.

The θ-burst stimulation of hippocampal slices from Wt mice perfused with L-serine at least 15 min before recordings led to an initial enhancement of the fEPSP slope followed by a decrease and stabilization period, reaching similar values to the Wt control condition (Wt: 50.6 ± 8.28, Wt + L-ser 10 μM: 35.3 ± 5.50, Wt + L-ser 50 μM: 34.4 ± 7.22, one-way ANOVA: F = 1.601, *p* = 0.229, Wt: *n* = 6, Wt + L-ser 10 μM: *n* = 8, Wt + L-ser 50 μM: *n* = 7, Figures 4A, C). In hippocampal slices from dnSNARE mice, where the SNARE-dependent release of gliotransmitters was selectively compromised, no changes were observed between hippocampal slices perfused or not with L-serine (dnSNARE: 33.06 ± 7.37, dnSNARE + L-ser 10 μM: 25.1 ± 9.80, dnSNARE + L-ser 50 μM: 29.6 ± 7.99, one-way ANOVA: F = 0.207, *p* = 0.815, dnSNARE: *n* = 6, dnSNARE + L-ser 10 μM: *n* = 8, dnSNARE + L-ser 50 μM: *n* = 7, Figures 4B, C).

Moreover, the presence of L-serine did not change the PTP for Wt mice (Wt: 112.9 ± 23.4, Wt + L-ser 10 μM: 89.6 ± 12.6, Wt + L-ser 50 μM: 93.2 ± 15.2, one-way ANOVA: F = 0.530, *p* = 0.598, Wt: *n* = 6, Wt + L-ser 10 μM: *n* = 8, Wt + L-ser 50 μM: *n* = 7, Figures 4A, D) nor for dnSNARE mice (dnSNARE: 61.5 ± 8.68, dnSNARE + L-ser 10 μM: 80.8 ± 12.9, dnSNARE + L-ser 50 μM: 88.9 ± 12.5, one-way ANOVA: F = 1.263, *p* = 0.307, dnSNARE: *n* = 6, dnSNARE + L-ser 10 μM: *n* = 8, dnSNARE + L-ser 50 μM: *n* = 7, Figures 4B, D). Thus, these results demonstrate that L-serine does not impact LTP, contrary to D-serine.

### 3.9 L-serine does not alter short forms of synaptic plasticity

PPF was assessed to confirm that L-serine does not modulate short-term forms of plasticity, by eliciting two consecutive fEPSPs (fEPSP1/fEPSP0). When comparing the ratio between the second and first fEPSP slope values (fEPSP1/fEPSP0) with and without L-serine, no differences were detected in hippocampal slices from Wt (Wt: 1.68 ± 0.06, Wt + L-ser 10 μM: 1.47 ± 0.05, Wt + L-ser 50 μM: 1.57 ± 0.05, one-way ANOVA: F = 2.120, *p* = 0.160, Wt: *n* = 4, Wt + L-ser 10 μM: *n* = 7, Wt + L-ser 50 μM: *n* = 5, Figure 4E) and neither in dnSNARE mice (dnSNARE: 1.59 ± 0.06, dnSNARE + L-ser 10 μM: 1.54 ± 0.13, dnSNARE + L-ser 50 μM: 1.48 ± 0.08, one-way ANOVA: F = 0.374, *p* = 0.694, dnSNARE: *n* = 4, dnSNARE + L-ser 10 μM: *n* = 6, dnSNARE + L-ser 50 μM: *n* = 7, Figure 4E) regardless L-serine concentration. Therefore, these results reinforce the previous data that demonstrated that D-serine nor L-serine interfere with presynaptic mechanisms associated with the release of neurotransmitters to the synapse.

### 3.10 L-serine affects basal synaptic transmission

To assess the influence of L-serine (that will be converted into D-serine) upon basal synaptic transmission, I/O curves were recorded from acute hippocampal slices perfused with and without L-serine, prepared from Wt and dnSNARE mice. In hippocampal slices from Wt mice, L-serine affected I/O curves profile (Wt: 1.78 ± 0.14, Wt + L-ser 10 μM: 1.47 ± 0.15, Wt + L-ser 50 μM: 1.41 ± 0.04, one-way ANOVA: F = 2.203, *p* = 0.150, Wt: *n* = 6, Wt + L-ser 10 μM: *n* = 6, Wt + L-ser 50 μM: *n* = 4, Figure 4F). Furthermore, L-serine affected in a similar fashion basal synaptic transmission in hippocampal slices of dnSNARE mice (dnSNARE: 2.57 ± 0.22, dnSNARE + L-ser 10 μM: 1.30 ± 0.12, dnSNARE + L-ser 50 μM: 1.22 ± 0.05, one-way ANOVA: F = 27.830, ****p = 0.000, dnSNARE: *n* = 6, dnSNARE + L-ser 10 μM: *n* = 4, dnSNARE + L-ser 50 μM: *n* = 7, Figure 4G). These results indicate that astrocyte-released D-serine holds a distinct and specific role in the regulation of LTP, while D-serine from other sources plays a modulatory role in the context of basal synaptic transmission.

## 4 Discussion

The perception of the role of astrocytes in brain function is a highly debated topic. There is growing evidence that astrocytes can receive, integrate, and respond to neural activity, by increasing the metabolic support of neurons and modulating synaptic transmission through the release of gliotransmitters. This process involves Ca^2+^-mediated signaling, ultimately leading to SNARE-dependent exocytosis (Pascual et al., 2005; Halassa et al., 2009; Araque et al., 2014; Lalo et al., 2014). In 2005, the dnSNARE mouse model was described (Pascual et al., 2005) as a model to elucidate the importance of vesicular gliotransmission. This model expresses the dominant-negative domain (cytosolic portion) of vesicular SNARE protein VAMP2/synaptobrevin2, in which the redundant expression of dnSNARE competes with endogenous VAMP2, compromising astrocytic exocytosis (Pascual et al., 2005). Nonetheless, Fujita and collaborators questioned the mouse model validity and suggested that neuronal populations may also express small quantities of the transgene, and thus results obtained with this mouse line may account for neuron-specific, rather than astrocyte-specific effects (Fujita et al., 2014). Hence, we performed complementary sets of experiments to validate the dnSNARE model as a good model to dissect the role of astrocyte-derived signaling, and therefore emphasize the evidence for the role of vesicular gliotransmitter release in astrocyte-neuron interactions, with a specific focus on the role of D-serine.

To confirm the specificity of astrocytes and rule out the neuronal expression of dnSNARE transgenes, we conducted immunostainings using markers for astrocytes (GFAP) and neurons [β-III tubulin, that stain neuronal cell bodies, dendrites, axons, and axonal termination (Geisert and Frankfurter, 1989; Figures 1C–E]. Additionally, we took advantage of the inherent fluorescence of the dnSNARE reporter (EGFP) to aid in our analysis. This approach allowed us to accurately identify and distinguish between which cells express the dnSNARE transgene. Our results do not support Fujita et al. (2014) conclusions that demonstrate that most NeuN-positive neurons in the cortex and hippocampus also exhibit low to moderate levels of EGFP. In our results, the typical astrocyte bushy morphology is observed in EGFP-labeled structures, similar to previous studies (Pascual et al., 2005; Sultan et al., 2015; Sardinha et al., 2017; Lalo et al., 2021). In addition, the EGFP strongly co-localizes with the astrocytic marker GFAP and fails to show colocalization with the neuronal marker (as also previously shown by Sardinha et al., 2017).

Furthermore, it was previously reported that EGFP staining and relative levels are correlated with dnSNARE expression, since mice that express higher levels of dnSNARE, also express higher levels of EGFP mRNA, which in turn translates into increased EGFP levels in those mice (Sardinha et al., 2017). Thus, we screened by western blot the transgenic protein levels in the hippocampus (brain region analyzed in the electrophysiology experiments), based on relative reporter EGFP levels. We observed that EGFP has a variable expression in the hippocampus, across dnSNARE mice (Figure 1B). The variable transgene expression observed among different mice may be caused by biological factors that affect gene regulation, for instance, the epigenetic state of a gene, which sums from the DNA methylation status, nucleosome assembly, and posttranslational histone modifications (De Jong et al., 2019).

Due to the influence of the dnSNARE protein expression on the level of exocytosis blockade (Sardinha et al., 2017), there is expected variability in dnSNARE mice results. This variability may explain the diverse LTP magnitudes observed and why some dnSNARE mice display similar results to Wt mice (Figures 2A–C. Notably, dnSNARE mice exhibit significantly smaller LTP magnitudes in comparison to Wt mice, indicating a role for astrocytes in regulating the strength of synaptic plasticity. This LTP impairment aligns with previous findings on the major role of astrocytes in the release of glutamate (Zhang et al., 2004) and D-serine (Sultan et al., 2015), which can modulate synaptic plasticity by activating NMDARs and enhancing LTP induction (Pascual et al., 2005). Additionally, other gliotransmitters can also modulate LTP by different pathways, as is the case of ATP, which can also be released by SNARE-dependent mechanisms (Lalo et al., 2014).

Basal synaptic transmission is fundamental for information processing in the CNS. It corresponds to the most elementary form of communication between neurons, involving the release of neurotransmitters at individual synapses in response to single action potentials. Our findings demonstrate that impairing gliotransmission enhances basal synaptic transmission (Figure 2F). In the I/O recordings, we measured the fEPSP slope, which is a measurement of how many receptors are bound by neurotransmitters, and is a good method of evaluation for the efficiency of the signaling mechanism at the postsynaptic side. In the present study, we observed an increase in basal synaptic transmission in mice with impaired vesicular release of gliotransmitters. These results align with the initial observations in the dnSNARE mice model (Pascual et al., 2005).

In dnSNARE mice, both PTP and PPF (Figures 2C, E, respectively), which are considered short-term forms of synaptic plasticity remained unaffected. As already described, both PPF and PTP are related to the release of neurotransmitters and are typically associated with Ca^2+^ increase in the presynaptic neuron (Korogod et al., 2007), providing valuable insights into the functionality of presynaptic mechanisms. The lack of differences in PPF and PTP suggests that gliotransmitters released by exocytosis predominantly impact synaptic transmission and plasticity at the postsynaptic level. Taken together, our results with existing literature, strongly support the critical role of astrocytic function and gliotransmission in maintaining synaptic plasticity and transmission in physiological conditions.

To date, there is no consistent evidence that definitively identifies whether astrocytes (Henneberger et al., 2010) or neurons (Benneyworth et al., 2012) serve as the primary source of extracellular D-serine. Therefore, we aimed to clarify astrocytes’ contribution to D-serine-mediated NMDAR signaling. Considering that the substrate for D-serine production, L-serine, in synthesized *de novo* in astrocytes, which express all the enzymes required to convert glucose into L-serine, we perfused hippocampal slices from dnSNARE mice with the compromised vesicular release of gliotransmitters, and respective controls, with D-serine and L-serine.

We hypothesized that if D-serine release relied on astrocytic integrity, and thus, perfusing dnSNARE hippocampal slices with D-serine and L-serine would yield different outcomes. Our results support this idea, as we found that D-serine increases LTP magnitude in hippocampal slices from mice with compromised gliotransmission (Figures 3A–D. However, this effect was not observed with L-serine perfusion, regardless of L-serine concentration (Figures 4A–C. We tested both the same concentration as D-serine (10 μM), and 50 μM, which is a concentration known to rescue LTP in animals lacking PHGDH in astrocytes, the enzyme required for the synthesis of L-serine from glucose (Le Douce et al., 2020)). Our findings suggest that after L-serine perfusion, D-serine is produced but cannot be released in hippocampal slices from mice with impaired SNARE-dependent gliotransmission to support LTP induction. Moreover, these results align with the “Serine shuttle” model (Wolosker, 2011), which proposed that L-serine synthesized by astrocytes is shuttled to neurons to fuel the synthesis of D-serine. Once released by neurons, D-serine is taken up by astrocytes. While the mechanism of D-serine release from each cell type is still a topic of debate, our results suggest that in the hippocampus, after being produced in neurons, D-serine shuttles to astrocytes, where it accumulates in glial vesicles to be released in a SNARE-dependent manner. This is further supported by the direct measurements of its extracellular levels of D-serine with microelectrode biosensors in the neocortex of dnSNARE mice (Lalo et al., 2018). Although the experiment conducted by Lalo and collaborators was focused on the neocortex, together with our results strongly suggests that astrocytes as the main source of D-serine to support LTP establishment.

Moreover, after D- and L-serine perfusion, there is a decrease in basal synaptic transmission in hippocampal slices dnSNARE animals (Figures 3G, 4G). In Wt animals, L- and D-serine do not affect the mechanisms underlying LTP, but exclusively modulate basal transmission, potentially by different pathways, leading to a dissociation between basal transmission and LTP. In the context of dnSNARE mice, the basal synaptic transmission decreases to levels comparable to the Wt group in presence of both L- and D-serine. This directly suggests that the observed enhancement in basal synaptic transmission in hippocampal slices of dnSNARE animals resulted from the absence of D-serine released by astrocytes. Studies by Pascual and colleagues in demonstrated that astrocytes play a role in controlling the extent to which a synapse can exhibit plasticity by suppressing excitatory transmission (Pascual et al., 2005). Additionally, during the induction LTP, astrocyte-derived adenosine was shown to depress neighboring unstimulated pathways. In contrast, recent research in the hippocampus has identified a subgroup of glutamatergic astrocytes that selectively express machinery resembling that of synaptic glutamate release (de Ceglia et al., 2023). Here, it was observed that the magnitude of θ -burst-evoked LTP was significantly lower in the synaptic fields containing astrocytes with Slc17a7 gene deletion (VGLUT1-GFAP-KO) when compared to Wt. It’s essential to note that the dnSNARE mice and VGLUT1-GFAP-KO mice are not the same model, and even though they have distinct purposes, they can be considered complementary in the sense that they shed light on different aspects of synaptic regulation. The coexistence of decreased LTP in both models suggests a potential common regulatory mechanism that impacts synaptic plasticity.

It’s crucial to acknowledge the complex nature of synaptic regulation, and that involves multiple neurotransmitters and cellular processes. A decreased astrocyte glutamate release could also lead to decreased glutamate levels, potentially enhancing GABAergic transmission and consequently decreasing synaptic transmission. Besides, it’s reasonable to hypothesize that mice lacking dnSNARE mice may develop compensatory mechanisms to counterbalance the reduced glutamate levels, possibly leading to increased basal synaptic transmission, as observed in our study. Overall, the observed decrease in basal synaptic transmission by D-serine might indicate the initiation of an unknown signaling cascade triggered by D-serine. This signal could be received by astrocytes, promoting morphological changes [such as an increase in size (Viana et al., 2023)] or feedback mechanisms between the “D-serine shuttle” and AMPA receptors (Gong et al., 2007; Ma et al., 2014), which in turn tightly modulate synaptic transmission.

Nevertheless, studies emphasizing the role of neurons in this pathway (Benneyworth et al., 2012) do not deny the ability of astrocytes to accumulate D-serine. Therefore, this theory continues to be compatible with our findings, highlighting the cooperation between neurons and astrocytes in governing D-serine dynamics. Overall, our data fills substantial gaps in understanding the mechanism of D-serine release: (1) it elucidates the predominant role of astrocytes in D-serine release, (2) demonstrates the crucial participation of astrocyte-derived D-serine in NMDAR-mediated synaptic plasticity, and (3) highlights the essential regulation of D-serine levels to uphold optimal basal synaptic transmission.

## Data availability statement

The raw data supporting the conclusions of this article will be made available by the authors, without undue reservation.

## Ethics statement

The animal studies were approved by the ORBEA - Instituto de Medicina Molecular João Lobo Antunes and Direção-Geral de Alimentação e Veterinária. The studies were conducted in accordance with the local legislation and institutional requirements. Written informed consent was obtained from the owners for the participation of their animals in this study.

## Author contributions

DA: Conceptualization, Investigation, Methodology, Writing – original draft, Writing – review & editing. JG: Investigation, Methodology, Writing – review & editing. FFR: Investigation, Methodology, Writing – review & editing. MD: Supervision, Writing – review & editing. AMS: Writing – review & editing. SHV: Conceptualization, Funding acquisition, Methodology, Supervision, Writing – original draft, Writing – review & editing.

## Supplementary material

Supplementary Figure 1 Uncropped images from Western blots. Uncropped Western blot images that correspond to Figure 1. Western blots of hippocampal slices tissue samples from dnSNARE and Wt mice depict immunoreactive bands for GFP (27 kDa) and GAPDH (loading control; 37 kDa). **(A)** Membrane with the labeling of GAPDH at the top and GFP at the bottom, demonstrating the presence and relative positions of these specific proteins. **(B)** Quantification of protein levels in each lane of Supplementary Figure 1A. **(C)** Another membrane with the labeling of GAPDH at the top and GFP at the bottom, similar to Supplementary Figure 1A. **(D)** Quantification of the bands observed in sSupplementary Figure 1C. **(E)** Protein marker that indicated the size of GAPDH and GFP. **(F)** The portion shown in Figure 1B was taken from the region highlighted by the red rectangle in Supplementary Figure 1A.

Supplementary Figure 2 **(A)** Time course of changes in fEPSP slope after θ-burst stimulation in hippocampal slices from Wt (Dox +) (*n* = 5) and dnSNARE mice (Dox +) (*n* = 5). Representative traces of fEPSPs before (dashed line) and after (bold line) stimulation are displayed above. Scale bar: 5 ms (horizontal), 0.5 mV (vertical). The blue highlighted column represents LTP (B), while the gray highlighted column corresponds to PTP (C). **(B)** Comparison of the magnitude of LTP induced by the θ-burst stimulation. **(C)** Comparison of PTP magnitude. **(D)** I/O curves showing how fEPSP slope changes with different stimulation intensities (60−320 μA) in hippocampal slices from Wt (Dox +) (*n* = 4) and dnSNARE mice (Dox +) (*n* = 4). All values are presented as mean ± S.E.M., with non-linear fittings of *n* independent experiments. Statistical significance was assessed by unpaired *t*-test.
