# Supplementary figures and images for: Astrocytes control hippocampal synaptic plasticity through the vesicular-dependent release of D-serine
Source: Front Cell Neurosci. 2023 Dec 7;17:1282841. doi: 10.3389/fncel.2023.1282841 (PMC10740624; doi:10.3389/fncel.2023.1282841)

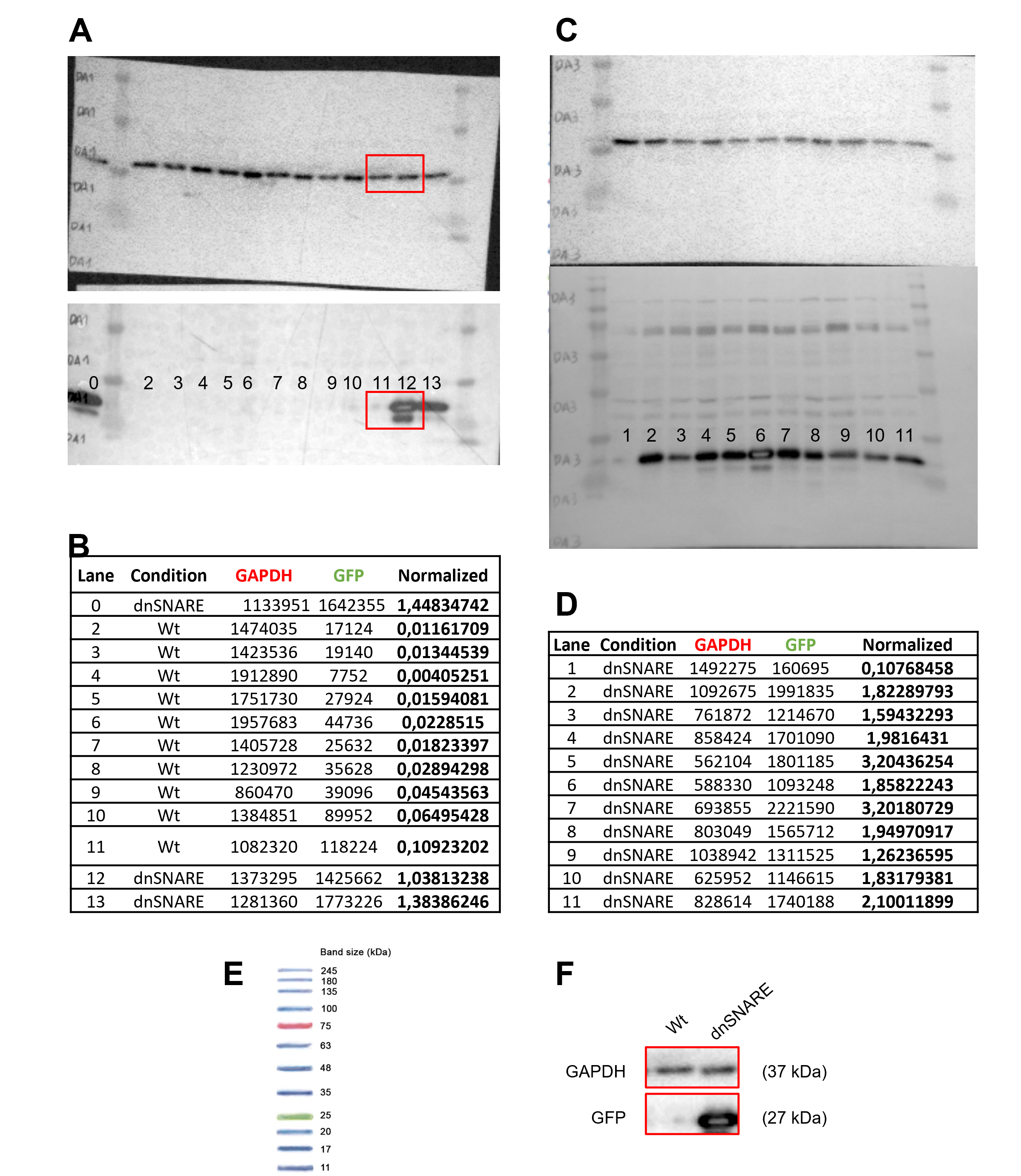

Supplement: Supplementary Figure 1 — Uncropped images from Western blots. Uncropped Western blot images that correspond to Figure 1. Western blots of hippocampal slices tissue samples from dnSNARE and Wt mice depict immunoreactive bands for GFP (27 kDa) and GAPDH (loading control; 37 kDa). (A) Membrane with the labeling of GAPDH at the top and GFP at the bottom, demonstrating the presence and relative positions of these specific proteins. (B) Quantification of protein levels in each lane of Supplementary Figure 1A. (C) Another membrane with the labeling of GAPDH at the top and GFP at the bottom, similar to Supplementary Figure 1A. (D) Quantification of the bands observed in sSupplementary Figure 1C. (E) Protein marker that indicated the size of GAPDH and GFP. (F) The portion shown in Figure 1B was taken from the region highlighted by the red rectangle in Supplementary Figure 1A. [file Image_1.TIF]

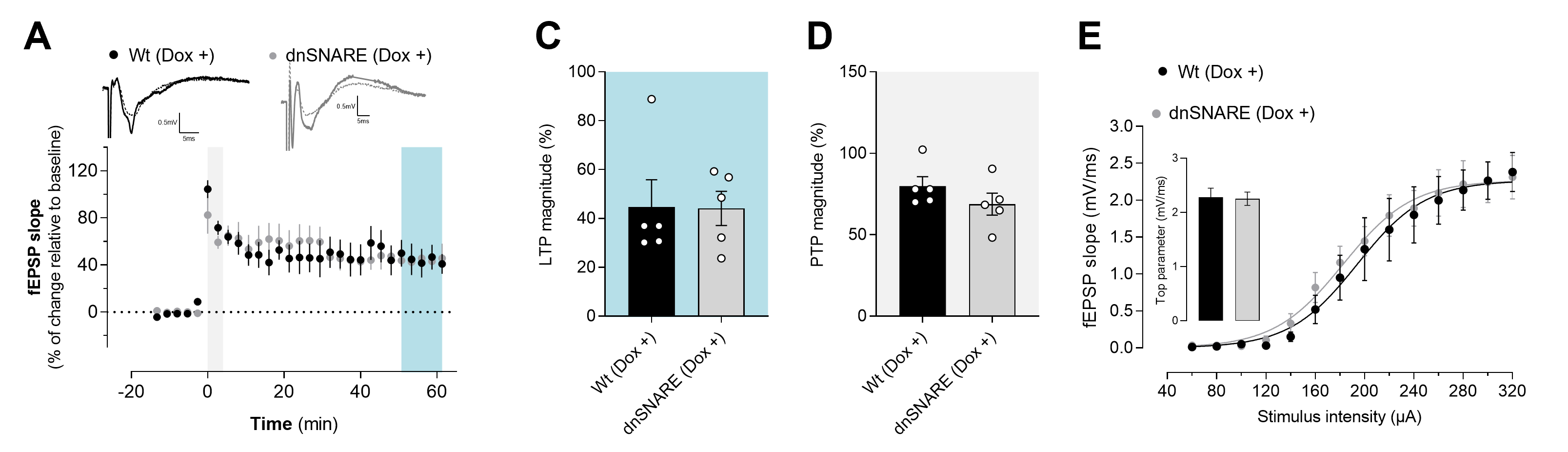

Supplement: Supplementary Figure 2 — (A) Time course of changes in fEPSP slope after θ-burst stimulation in hippocampal slices from Wt (Dox +) (n = 5) and dnSNARE mice (Dox +) (n = 5). Representative traces of fEPSPs before (dashed line) and after (bold line) stimulation are displayed above. Scale bar: 5 ms (horizontal), 0.5 mV (vertical). The blue highlighted column represents LTP (B), while the gray highlighted column corresponds to PTP (C). (B) Comparison of the magnitude of LTP induced by the θ-burst stimulation. (C) Comparison of PTP magnitude. (D) I/O curves showing how fEPSP slope changes with different stimulation intensities (60−320 μA) in hippocampal slices from Wt (Dox +) (n = 4) and dnSNARE mice (Dox +) (n = 4). All values are presented as mean ± S.E.M., with non-linear fittings of n independent experiments. Statistical significance was assessed by unpaired t-test. [file Image_2.TIF]
